# Severe weight loss in a hypothyroid patient as an acute presentation of autoimmune polyglandular syndrome type II

**DOI:** 10.1007/s42000-021-00344-9

**Published:** 2022-02-19

**Authors:** Elvira Silajdzija, Sofie Bliddal, Line Borgwardt, Maria Rossing, Anne Jarløv, Claus Henrik Nielsen, Ulla Feldt-Rasmussen

**Affiliations:** 1 Faculty of Health and Medical Research, Copenhagen University, Copenhagen, Denmark; 2 Department of Medical Endocrinology and Metabolism, Rigshospitalet, Copenhagen University, Copenhagen, Denmark; 3 Institute of Inflammation Research, Center for Rheumatology and Spine Diseases, Rigshospitalet, Copenhagen University, Copenhagen, Denmark; 4 Center for Genomic Medicine, Rigshospitalet, Copenhagen University, Copenhagen, Denmark

**Keywords:** Autoimmune polyglandular syndrome type II, HLA, Hypothyroidism, Autoimmunity, Addison’s disease, Diabetes

## Abstract

**Background:**

Autoimmune disease, including autoimmune thyroid disease, with uncharacteristic symptoms can be due to additional severe disease. We report a life-threatening debut of autoimmune polyglandular syndrome type II (APS II) defined as Addison’s disease combined with autoimmune diabetes and/or thyroid disease.

**Patient findings:**

A 33-year-old male with newly diagnosed hypothyroidism was referred to a tertiary center due to fatigue and 20-kg rapid weight loss. Malignancy was excluded. After a gastroscopy, he developed Addison’s crisis; he was admitted to our hospital and stabilized. Final diagnoses included Hashimoto’s thyroiditis, Addison’s disease, vitiligo, and pernicious anemia. Whole genome sequencing found no genetic variants associated with component diseases. Human leukocyte antigen typing revealed DR3/DR4 and DQ8/DQ2 heterozygosity associated with APS II.

**Summary:**

A patient with Hashimoto’s thyroiditis and weight loss presented with Addison’s crisis and was diagnosed with APS II.

**Conclusions:**

Awareness of potential polyautoimmunity in clinical evaluation of patients with thyroid disease improves diagnosis and can be lifesaving.

## Introduction

In 1926, Martin Benno Schmidt described the association of Addison’s disease and chronic lymphatic thyroiditis and named it “Schmidt’s syndrome.” Now more commonly referred to as autoimmune polyglandular syndrome type II (APS II), the syndrome is defined by the presence of Addison’s disease together with diabetes mellitus type I and/or autoimmune thyroid disease [1] and occurs in approximately 1 per 20,000 inhabitants [2]. The cause is likely multifactorial including a genetic predisposition [1, 2].

Uncharacteristic symptoms presenting in patients with an autoimmune disease can be a sign of additional underlying severe disease. We present a case of hypothyroidism and severe weight loss as a reminder that polyautoimmunity is potentially a life-threatening phenomenon.

## Patient

A 33-year-old male consulted his general practitioner due to severe fatigue and an unintended weight loss of 20 kg over 3 months. Apart from suffering from vitiligo since childhood, the patient reported being in good health. He fathered two children without need of fertility treatment.

### Family history

The patient’s father had, like his son, suffered from vitiligo since childhood. The patient’s mother died of liver cancer in her 60s. The patient had one half-sister on his mother’s side. Both the mother and the half-sister had rheumatoid arthritis. There were no other known diseases (including thyroid disease, diabetes, and other endocrine diseases) among the relatives.

### Initial work-up

The patient’s general practitioner measured his thyroid function and diagnosed the patient with autoimmune hypothyroidism (positive thyroid peroxidase antibodies and thyroid stimulating hormone (TSH) of 44 mIU/L). Due to his weight loss, the patient was referred to a tertiary referral center on suspicion of malignancy. The clinical examination found no signs of tumors or lymphadenopathy, chest X-ray was normal, and an abdominal ultrasound scan showed slight steatosis but was otherwise normal. The possibility of polyautoimmunity was explored by referral to the gastrointestinal unit and the endocrine unit. A gastroscopy revealed signs of autoimmune gastritis, which was later confirmed by histological examination of ventricular biopsies. The patient subsequently reported having been unable to get out of bed in the days following the gastroscopy as well as being extremely tired, nauseous, and unable to eat or drink.

### Endocrine unit

Three days after the gastroscopy, the patient was brought to the tertiary endocrine out-patient clinic, where a Synacthen® test revealed a critically low 30-min p-cortisol of 33 nmol/L ((1.2 µg/dL), reference ≥ 420 nmol/L (15.22 µg/dL)). He was immediately admitted as an in-patient and treated for Addison’s crisis according to standard of care.

**Fig. 1:**
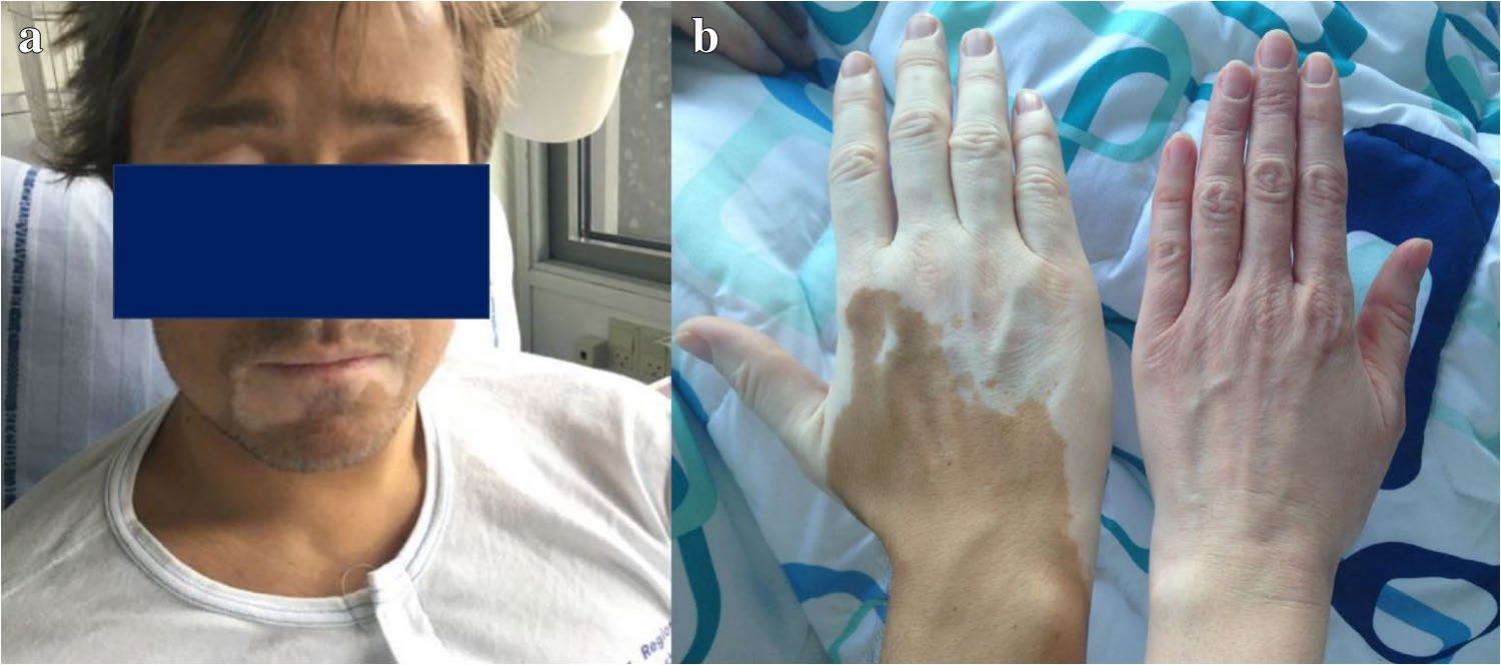
**A**, **B** Hypopigmentation and hyperpigmentation in a patient with hypothyroidism. The patient had characteristic physical appearance with both hypopigmentation due to his vitiligo and hyperpigmentation due to excessive ACTH production preceded by the cleavage of the prohormone, pro-opiomelanocortin (POMC) leaving melanocyte-stimulating hormone as a by-product (**A**). This was especially visible when compared with a standard Caucasian Danish winter tan (right-hand picture, right hand) (**B**). Pictures published with permission from the patient

Physical examination showed poor general appearance, white patches on multiple areas of the skin, hyperpigmentation in the remaining skin areas (Fig. 1A and 1B), and slight activity-related dyspnea. The patient’s blood pressure was 74/69 mmHg upon admission. Acute administration of iv. hydrocortisone and isotonic saline enabled stabilization.

The patient was diagnosed with Addison’s disease, Hashimoto’s thyroiditis, vitiligo, and pernicious anemia (and high-level anti-glutamate decarboxylase 65 autoantibodies) consistent with APS II. His condition markedly improved following treatment with hydrocortisone, fludrocortisone, hydroxycobalamine, and levothyroxine substitution. Table 1 depicts results from blood tests upon admission to the endocrine unit and 1 year later.

**Table 1:** Laboratory tests at time of admission and at 1-year follow-up

Test	At admission	At 1-year follow-up
P-sodium (137–144 mmol/L)	124	142
ACTH (2–11 pmol /L)	280	12
TSH (0.4–4.8 mIU/L)	62	6.39
Total T4 (70–140 nmol/L)	68	85
Free T4 (12–22 pmol /L)	8.2	17.1
Hemoglobin (8.3–10.5 mmol/L)	7.1	NA
Vitamin B12 (> 200 pmol /L)	98	303
HbA1c (< 48 mmol/mol)	35	34

Standard blood work-up at time of admission and at 1-year follow-up. Further tests showed high concentrations of antibodies towards thyroid peroxidase, the cortical adrenal gland, gastric parietal cells, and glutamate decarboxylase 65. Reference intervals appear in the parentheses

Abbreviations: *ACTH*, adrenocorticotropic hormone; *HbA1c*, hemoglobin A1c; *NA*, not available; *T4*, thyroxine; *TSH*, thyroid stimulating hormone

### Follow-up

Over 1 year of follow-up, the patient experienced increasing well-being and was able to resume his job within a couple of months from diagnosis.

### Genetic analyses

The patient and his father went through whole genome sequencing (WGS) and a CytoScan HD array analysis to screen for potential pathogenic variants and copy number alterations, respectively. No pathogenic variants or copy number alterations associated with the phenotype were identified by genomic screening despite a thorough search including genetic components involved in immunoregulation (i.e., cytotoxic T-lymphocyte-associated antigen-4 (CTLA-4), protein tyrosine phosphatase, non-receptor type 22 (PTPN22), and signal transducer and activator of transcription 4 (STAT4)).

HLA typing was performed by next generation sequencing (NGS) and revealed the following HLA haplotypes: HLA-A*03:01-B*07:02-C*07:02-DRB1*04:01:01G-DQB1*03:02-DPB1*02:01 and HLA-A*01:01-HLA-B*08:01-C*07:01-DRB1*03:01:01G-DQB1*02:01-DPB1*04:01.

## Discussion

APS II is a rare, but potentially life-threatening disease, as seen in the presented case. In general, there is an increased risk of development of other autoimmune disorders in patients with autoimmune endocrinopathies, of which thyroid disease is the most common [3–5]. Clinical awareness of this fact can save lives, specifically, by performing a corticotropin stimulation test for Addison’s disease prior to other examinations and treatments, in order to minimize the risk of Addison’s crisis. Furthermore, the various phenotypic presentations of the diseases of the syndrome will define the clinical challenges in individual patients. Accordingly, for example, pernicious anemia can reduce the uptake of any oral treatment, thus necessitating increasing the treatment dose. This is often the case in patients treated with levothyroxine due to hypothyroidism. Autoimmune thyroid disease is the most common autoimmune disease, and is often an overlooked disease co-occurring with other autoimmune diseases (particularly in males) [6]. Other autoimmune diseases compromising gastrointestinal absorption include ulcerative colitis, celiac disease [7, 8], and systemic lupus erythematosus [9]. Failure to recognize autoimmune hypothyroidism or Addison’s disease can further result in severe hypoglycemia in type 1 diabetes mellitus.

The highly polymorphic human leukocyte antigen (HLA) molecules determine the recognition pattern of cytotoxic T cells, T-helper cells, and regulatory T cells. Specific genetic variants of the HLA region located on chromosome 6 have been associated with most autoimmune diseases as a risk factor for the loss of self-tolerance. Among the first studies of HLA types in APS II patients, haplotypes including HLA-A1 or HLA-B8 were shown to be more prevalent among patients than relatives without disease or healthy controls [10–13]. Later, the specification of HLA-DR3-DQ2 or DR4-DQ8 (serotype DQ2 corresponding to genotype (DQA1*0501)-DQB1*0201 and DQ8 to (DQA1*0301)-DQB1*0302) was convincingly demonstrated in several studies [14–17]. Myhre et al. [14] found the DR3-DQ2/DR4-DQ8 genotype in 29 of 74 (39.2%) of patients with Addison’s disease compared to 5 of 290 (1.7%) healthy controls (odds ratio 36.7, *p*-value < 0.001), and this was especially prevalent among APS II patients (10 of 12 patients had this haplotype). Huang et al. [18] also demonstrated the synergistic effect of the presence of this heterozygosity (DRB1*03-DQB1*0201/ DRB1*04-DQB1*0302) in seven of 17 (41%) APS II patients compared to five of 191 (3%) healthy controls (odds ratio 26, *p* < 0.001). Most recently, Frommer et al. [19] confirmed that APS II (and other subgroups of APS) differed significantly from controls in DQA1, DQB1, and DRB1 alleles (*p* < 0.0001). This included particularly haplotypes DRB1*03:01-DQA1*05:01-DQB1*02:01 and DRB1*04:01-DQA1*03:01-DQB1*03:02 (approx. 50% in APS II vs. 15% in controls [specific proportions not given]). Some of these studies suggested differences between various subtypes of APS II depending on the component diseases [18, 19]. HLA-DR3-DQ2/DR4-DQ8 has also been strongly associated with type 1 diabetes mellitus and celiac disease [20, 21]. A study of the general Danish population found 4.1% to be heterozygous for HLA-DQ2-DQ8 [22]. Overall, these findings match the haplotype of the patient in the present case report.

Although not so in the present case, several other genetic components involved in immunoregulation (i.e., CTLA-4, PTPN22, and STAT4) have also been associated with the different components of APS II [1, 23–26]. Recent advances in genetic testing, e.g., WGS, will likely expand our knowledge of possible genetic risk factors in APS II [27–29] and help describe the multivariant genetic character of the disease including differences between various subtypes. New genetic landmarks may thus offer a more personalized approach to patient care compared to what is currently possible by using only serological monitoring (Table 2).

**Table 2:** Serological testing for autoantibodies in patients with suspected polyglandular autoimmune syndrome type II

Autoantibodies against	Component disease
TPO	Hashimoto’s thyroiditis
GAD-65, IA-2, ZnT8	Type 1 diabetes
21-hydroxylase	Addison’s disease
Ovaries*, side-chain cleavage enzyme, 21-hydroxylase	Premature ovarian insufficiency
Intrinsic factor, H^+^K^+^ATPase, parietal cell	Autoimmune gastritis, pernicious anemia
Transglutaminase-2	Celiac disease

Overview of autoantibodies found in component diseases of polyglandular autoimmune syndrome type II. Adapted from Husebye et al. [26]

^*^Anti-ovarian antibodies consist of a sum of antibodies against ovarian tissue antigens and may differ between commercial assays

Abbreviations: *GAD-65*, glutamic acid decarboxylase-65; *IA-2*, islet antibody-2; *TPO*, thyroid peroxidase; *ZnT8*, zinc transporter 8

## Conclusion

As a reminder of a potentially life-threatening phenomenon, we report a case of polyautoimmunity presenting as hypothyroidism and severe weight loss developing into an Addison’s crisis. The patient was diagnosed with Hashimoto’s thyroiditis, Addison’s disease, vitiligo, and pernicious anemia (and high-level anti-glutamate decarboxylase 65 autoantibodies) consistent with APS II. Genetic testing demonstrated heterozygosity of HLA-DR3-DQ2 and DR4-DQ8, which have previously been associated with APS II.

Awareness of the possibility of polyautoimmunity in patients with autoimmune thyroid disease is important for establishing a clinical diagnosis and can be lifesaving, as seen in the present patient report.
